# Co-option of the same ancestral gene family gave rise to mammalian and reptilian toxins

**DOI:** 10.1186/s12915-021-01191-1

**Published:** 2021-12-23

**Authors:** Agneesh Barua, Ivan Koludarov, Alexander S. Mikheyev

**Affiliations:** 1 Ecology and Evolution Unit, Okinawa Institute of Science and Technology Graduate University, Okinawa, Japan; 2 Animal Venomics Group, Justus Leibig University, Giessen, Germany; 3 Research School of Biology, Australian National University, Canberra, ACT Australia

**Keywords:** Evolution, Venom, Phylogenetics, Kallikreins, Comparative genomics

## Abstract

**Background:**

Evolution can occur with surprising predictability when organisms face similar ecological challenges. For most traits, it is difficult to ascertain whether this occurs due to constraints imposed by the number of possible phenotypic solutions or because of parallel responses by shared genetic and regulatory architecture. Exceptionally, oral venoms are a tractable model of trait evolution, being largely composed of proteinaceous toxins that have evolved in many tetrapods, ranging from reptiles to mammals. Given the diversity of venomous lineages, they are believed to have evolved convergently, even though biochemically similar toxins occur in all taxa.

**Results:**

Here, we investigate whether ancestral genes harbouring similar biochemical activity may have primed venom evolution, focusing on the origins of kallikrein-like serine proteases that form the core of most vertebrate oral venoms. Using syntenic relationships between genes flanking known toxins, we traced the origin of kallikreins to a single locus containing one or more nearby paralogous kallikrein-like clusters. Additionally, phylogenetic analysis of vertebrate serine proteases revealed that kallikrein-like toxins in mammals and reptiles are genetically distinct from non-toxin ones.

**Conclusions:**

Given the shared regulatory and genetic machinery, these findings suggest that tetrapod venoms evolved by co-option of proteins that were likely already present in saliva. We term such genes ‘toxipotent’—in the case of salivary kallikreins they already had potent vasodilatory activity that was weaponized by venomous lineages. Furthermore, the ubiquitous distribution of kallikreins across vertebrates suggests that the evolution of envenomation may be more common than previously recognized, blurring the line between venomous and non-venomous animals.

**Supplementary Information:**

The online version contains supplementary material available at 10.1186/s12915-021-01191-1.

## Background

The extent to which shared history determines repeated evolution of traits remains an important and open question in evolutionary biology. Experiments replaying the tape of life showed that phenotypes can arise through a combination of deterministic forces like natural selection and stochastic, non-deterministic forces like mutation and genetic drift [1]. The historical nature of evolution gives it a certain degree of ‘contingency’, such that past events can drastically alter evolutionary trajectories [1]. The role of contingency and chance in shaping evolution is substantial, so much so that a single positive mutation might allow a genetic system to thrive and tolerate less favourable mutations or even create scenarios where similar selection pressures might not lead to the same evolutionary outcome [2, 3]. Therefore, tracing the evolutionary trajectory of genes can offer valuable information regarding the role of contingency and chance in shaping phenotypes. Selection on homologous and deeply conserved genetic mechanisms can repeatedly produce diverse phenotypes. For example, developmental toolkit genes regulate animal development and are involved in controlling differentiation among body axes, generating the extensive diversity in animal forms [4]. In plants, modifications of a shared developmental network have repeatedly led to the evolution of bilateral floral symmetry from a radially symmetric ancestor [5]. However, most traits are not controlled by such master regulators but emerge from complex interactions within polygenic networks. Yet, how regulatory complexity yields phenotypic novelty remains poorly understood.

To fully reveal the course of evolutionary changes, it is essential to have a good understanding of the link between genotype and the phenotype they produce [6–8]. But due to the complex nature of most biological traits, this link is rarely clear. Thus, while short-term evolution via quantitative genetic models is relatively easy to predict, how qualitatively novel traits arise repeatedly is less clear. Exceptionally, reptilian and mammalian oral venoms are proteinaceous cocktails where each constituent toxin can be traced to a specific locus, providing an unprecedented level of genetic tractability [9–12]. Venoms primarily evolve through sequence and gene expression changes of their constituent toxins, the phenotypic effects of which are clearly understood [10, 13–16]. Venoms are also excellent examples of convergent traits where individual toxins are believed to have been convergently recruited [11, 17, 18]. This high degree of convergence coupled with the genetic tractability of venom has allowed researchers to uncover genetic changes that contributed to the convergence of venom components, particularly in reptiles. For example, snake venom metalloproteinases (SVMP), which make up the primary component of viperid venoms, evolved through a series of deletions and tandem duplication from a single deeply conserved adam28 disintegrin [19]. Similarly, deletion and lineage specific expansion of phospholipase A2 (PLA2) lead to the evolution of novel venom phenotypes in some viperids [20, 21]. However, a similar tracing of genetic origins is still incomplete for the most ubiquitous toxin family in venom—the serine proteases.

Found in all kingdoms of cellular life as well as in viruses, serine proteases are perhaps the most widely distributed group of proteolytic enzymes [22]. Although best characterized in snakes, kallikrein-like (KLK-like) serine proteases are the main components in mammalian venom like that in *Blarina* shrews and *Solenodon*, as well as reptilian venoms in *Heloderma* lizards [11, 23, 24]. Yet, given the diversity of kallikrein types within and between organisms, researchers recognized early on that “the kallikreins from different sources are not identical molecules, as originally assumed” [17]. This view has persisted to the present day, and even within mammals, co-option of KLK-like serine proteases into venom is believed to represent convergence [11]. By contrast, Fry and colleagues hypothesized the recruitment of kallikreins into reptile and mammal venoms could have occurred from a phylogenetically common source [25, 26]. Yet, distinguishing these hypotheses has been difficult until now given the vast number of serine proteases found in vertebrate genomes. Phylogenetic studies have not yet adequately sampled genes from reptilian and mammalian taxa and their phylogenetic relationships remain unresolved [27, 28]. Specifically, Hargreaves et al. [29] noted that “the orthology of previously published Toxicoferan Kallikrein genes is currently unclear”.

Here, we benefit from recent advances in genomics, which allowed us to reconstruct syntenic relationships between KLK-like toxins and their flanking genes in order to correctly identify paralogs dating back to a common tetrapod ancestor. We were then able to use phylogenetics to resolve the evolutionary origins of venom KLK-like genes. Our results show that mammalian and reptilian venom serine proteases have an origin distinct from other non-venomous KLKs and have been recruited into venom in parallel. This is in line with previous results that the repeated evolution of venom in vertebrates has occurred due to exaptation of already existing components rather than independent evolution of the similar components in different lineages.

## Results

### Genomic organization of the snake-venom like (SVL) and KLK loci

To determine the genetic history of the venom KLK-like toxins, we identified homologues of the kallikreins in the genomes of mammals, reptiles, amphibians. We specifically focused on tissue kallikreins (TKLs) which are abundant in tissues like pancreas, kidney, as well as in saliva. They have functions ranging from mediating blood pressure and muscle contraction to inflammatory cascades and pain induction [28]. Since they are also the gene family associated with toxicity of various animal venoms we restricted ourselves to only TKLs [30]. Mammalian kallikrein toxins are closely related to the KLK1 gene [11], and we will refer to them as KLK-like toxins. The reptilian counterparts are highly syntenic to snake venom serine protease (SVSP) in vipers (Additional file 1: Fig. S1). Therefore, we refer to their reptilian counterparts as snake venom-like (SVL) toxins.

**Fig. 1:**
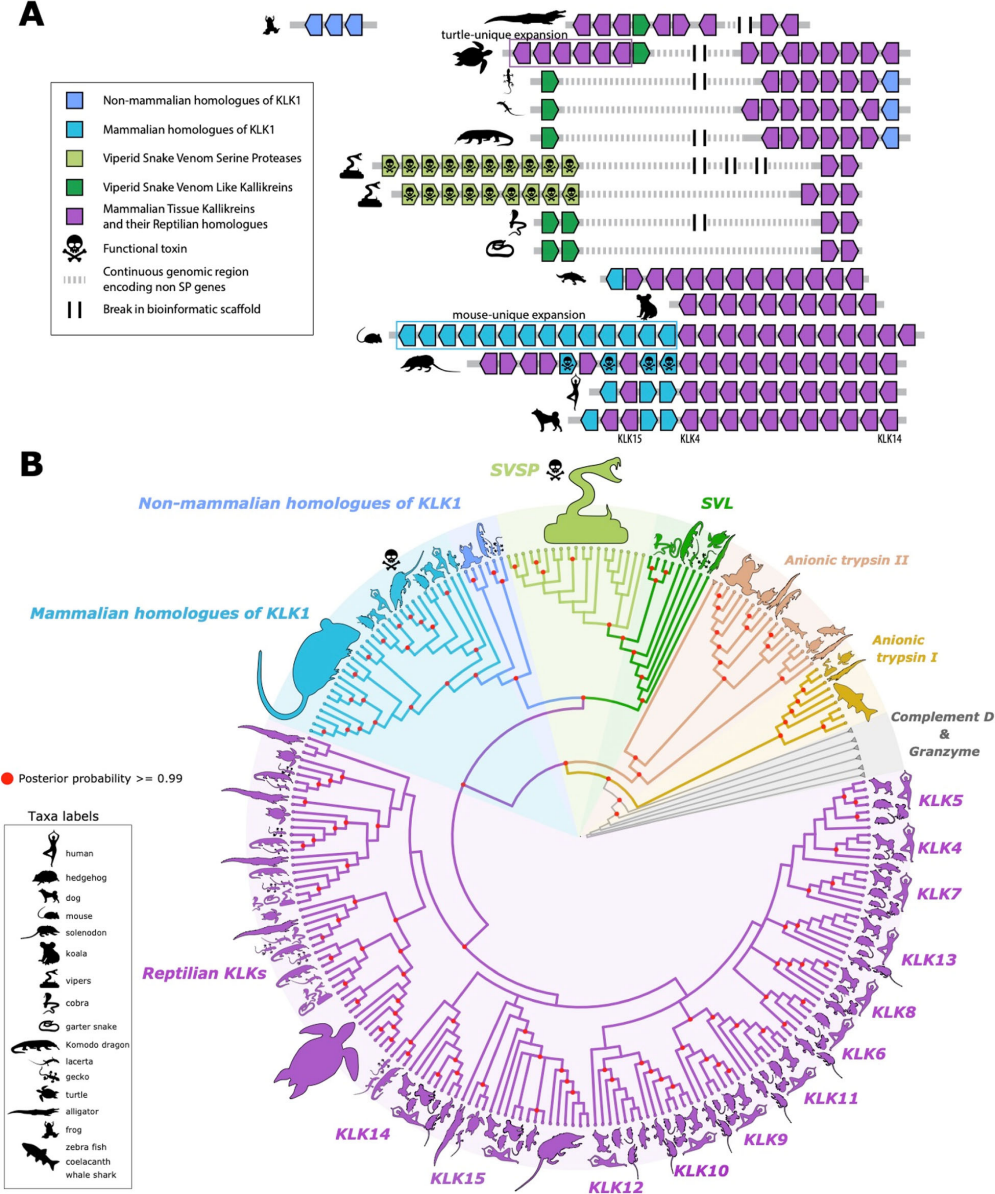
Origins and diversification of tissue kallikreins (TKL). **A** TKL genes are located at a single genomic locus. In mammals, TKL genes are found in a single cluster, but in reptiles, they are scattered across two to three nearby clusters located several hundred kilobases apart. Venom evolution is associated with expansions of toxin-containing gene clusters, but there are also lineage-specific expansions that are not linked to venom evolution (e.g. turtles and mice). In existing genomic assemblies, the TKL clusters are often fragmented (represented by dashed line) across different scaffolds, but they share many common genes and are clearly contiguous (Supplementary Figure 1 and 2). **B** Phylogenetic analysis revealed that tetrapod TKLs originated from a common ancestor with vertebrate anionic trypsins, which are commonly expressed in the pancreas and are found elsewhere in the genome. TKLs diverged into two distinct clades, one comprising the KLK4-KLK15 lineages and the other the KLK1/2/3-SVSP/SVL lineage that contains toxipotent genes. Species silhouettes represent members of entire clades rather than a strict node to species demarcation. For a more conventional format, please refer to phylogeny (Supplementary Figure 2 and supplementary dataset 1) in supplementary. Serine protease-based toxins are homologs deriving from the same ancestral gene, implying that these toxins originated in parallel venoms in reptiles and mammals

In humans, TKLs are located in a cluster comprising 15 copies (genes KLK1 through KLK15) on the 19th chromosome (19q13.4). TKL clusters are also found in other mammalian genomes, though the degree of synteny differs considerably. The KLK1 and KLK15 genes underwent tandem duplications in venomous mammals like solenodon and blarina [11, 31]. The expanded KLK1 genes contribute to the major toxin component of solenodon salivary and venomous secretions [11] (Fig. 1A). Unlike mammalian genomes, where KLK-like genes are contiguous, reptilian genomes have 2–3 gene clusters separated by several hundred kilobases and interrupted by other types of genes. One of these clusters contains genes that gave rise to viperid SVSPs (Fig. 1A). In highly venomous snakes like vipers, the expansion of snake venom serine protease (SVSP) genes is linked to the diversification of the venom phenotype [16, 32], paralleling expansions associated with the evolution of mammalian venoms. Thus, in both reptiles and mammals a single gene cluster gave rise to kallikrein-like serine protease toxins. However, the relationship between these genes is difficult to ascertain based on synteny alone and detailed phylogenetic analysis was needed.

### Phylogeny of SVL and mammalian KLK genes

We conducted phylogenetic analyses to better understand relationships between and with TKL genes and to identify the likely origin of these genes. Since the TKL-like genes represent a large and diverse gene family, they were essential that we sample a wide repertoire of genes across a wide taxonomic distribution. To do this, we searched for sequences closely related to KLKs in mammals, reptiles, amphibians, and fish, as classified by NCBI. NCBI’s classifications rely on a combination of calculated orthology and similarity in protein architectures based on sequences in the RefSeq database. This gene set included many non-KLK serine proteases like anionic trypsins, plasminogen, granzyme, and complement D, along with a list of all possible KLK-related sequences that are available in NCBI (with a combined total of a few thousand sequences). In order to isolate phylogenetically comparable genes, we used this large gene set (see the “Methods” section) as input for OrthoFinder. OrthoFinder classified genes into several large orthogroups. We isolated the orthogroup that contained TKL, SVL, and SVSP genes (Additional file 2) and resolved the phylogenetic relationship between genes within this group. This approach also allowed us to appropriately root our tree and reconstruct the early evolutionary history of TKLs.

We used a maximum-likelihood as well as a Bayesian approach to construct the phylogeny (see the “Methods” section). Both approaches yielded the same structure at each key nodes (discussed below) as well as comparable levels of support (Additional file 3 and Additional file 4). For the sake of brevity, we only display the Bayesian phylogeny (Fig. 1B) with Bayesian node supports at key nodes. Using complement D and granzyme (Fig. 1B; grey branches) as outgroups, we observed a clear origin of TKLs from two groups of anionic trypsins that are shared between reptiles, amphibians, and fish. After the divergence from anionic trypsins, the TKLs split into two separate lineages. While most of the mammalian KLK branching is consistent with previously published mammalian TLK phylogenies [27, 28], our tree has better overall support; for instance, in Koumandou et al. [28], the divergence of mammalian KLK1-KLK2-KLK3 (mKLK1,2,3, includes KLK toxins) has a Bayesian node support of ~ 0.80 whereas our trees have a support > 0.99. Additionally, we observe several new relationships between genes that were previously not described. First, the SVSP-SVL and mKLK1,2,3 genes formed a monophyletic clade sister to the other KLKs (Fig. 1B). This topology has high posterior probability (> 0.99) and was further supported by stepping-stone sampling (Bayes Factor of 111.0 in favour of monophyly between KLK1/2/3 and SVL-like genes vs. the monophy of all KLK-like genes excluding SVSP-like genes). Within the SVL-mKLK1,2,3 clade, the reptilian and mammalian genes form their own sub-clades. The SVL genes appear to group according to the toxicofera classification, with SVL in cobra (*Naja naja*) and garter snake (*Thamnophis sirtalis*) forming a sister clade to the SVSP in elapids and vipers, while non-toxicoferans like the leopard gecko (*Eublepharis macularius*) and the sand lizard (*Lacerta agilis*) forming individual lineages (Fig. 1B). Second, KLK15 and KLK14 in reptiles formed a clade with their mammalian homologs; however, several reptile KLKs formed separate reptile specific clades.

### Selection analysis of SVL and mammalian KLK genes

The SVL genes in reptiles are homologous to SVSPs and could have a potential role in imparting toxicity to salivary secretions, as suggested for example in Anguimorph lizards [24]. Under this assumption, we would expect selection to vary in species believed to have toxic oral secretions, i.e. species belonging to the clade Toxicofera, as compared to non-toxicoferans. To test the toxicofera hypothesis, we performed branch selection analysis using Phylogenetic Analysis by Maximum Likelihood (PAML) [33]. We applied a ‘free ratio’ model for branches leading up to toxicofera and compared its fit to a uniform ‘one ratio’ model for all branches. For a better representation of the toxicofera clade, we obtained additional anguimorpha kallikrein sequences from NCBI. We only included coding sequences that encoded for a mature protein and formed a monophyletic clade with our already identified SVL genes (Additional file 1: Fig.S4). We did not include venomous snakes in our test because higher selection for toxin genes in venomous snakes is already an established fact and could bias analyses [10, 34, 35]. The two-rate model fits significantly better (likelihood ratio test (LRT), *p* < 0.001) than the uniform one rate model suggesting that toxicoferan SVL genes experienced different selective pressures as compared to non-toxicoferans. We performed the same analysis to test whether venomous mammals experienced different selection as compared to non-venomous ones. We use the KLK toxins in *Solenodon* genes and their homologs in humans, dogs, and hedgehogs. The branches leading up to venomous mammals *Solenodon* and *Blarina* experienced selective forces significantly different from the rest of the tree (LRT, *p* < 0.001). While it is difficult to attribute positive selection as the reason for differences in selective pressures from this simple test, some branches (both in toxicofera and venomous mammals) did show high *ω* values (> 1) that are indicative of positive diversifying selection (Additional file 5 and 6). To get a better picture of the selective forces driving the evolution of the toxicofera and venomous mammals’ clade, we performed several branch-specific tests using the Datamonkey server [36].

**Fig. 2:**
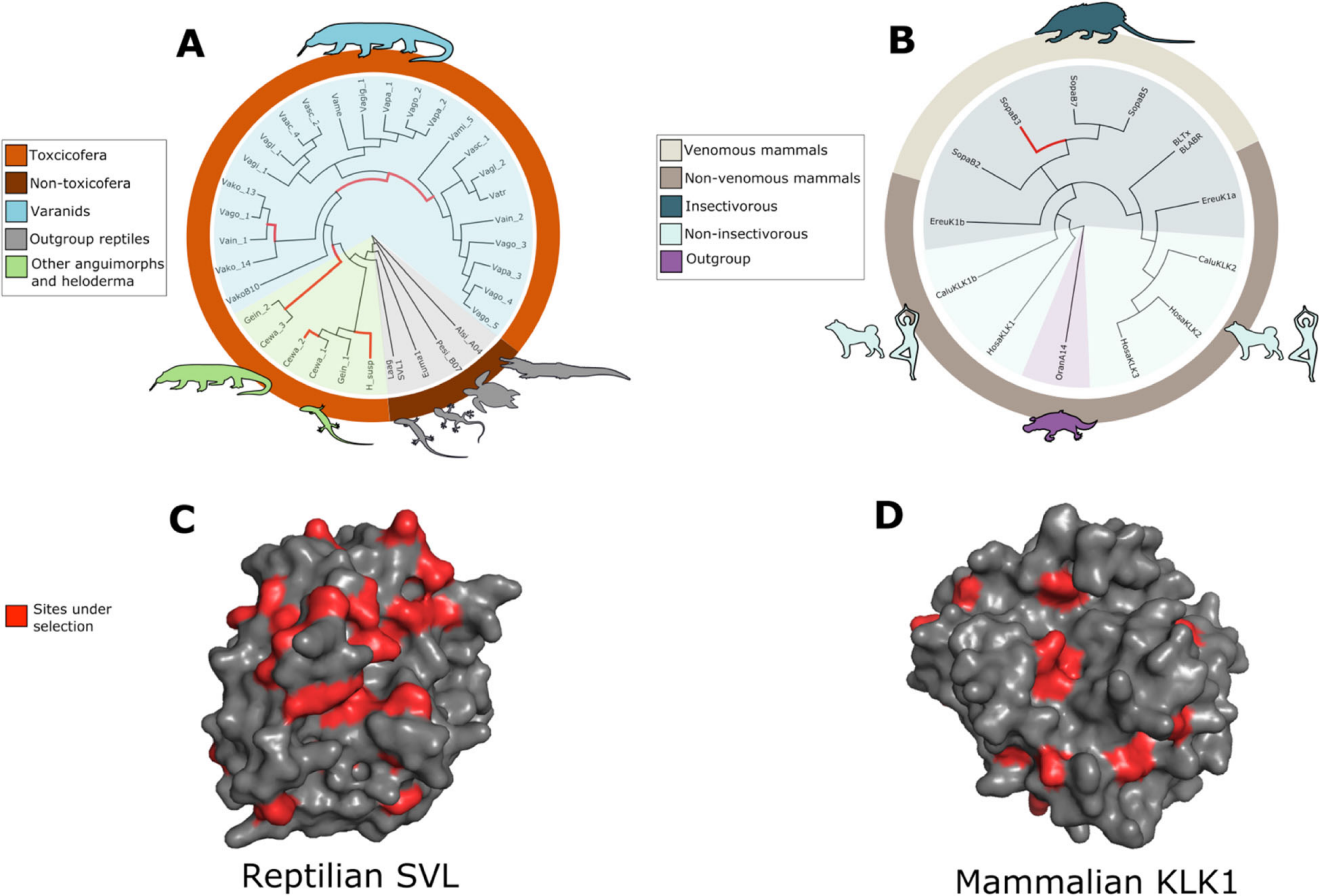
Venomous lineages experienced different selective forces as compared to non-venomous ones. **A** Toxicofera experienced different selection as compared to non-toxicoferan reptiles. aBSREL found evidence for diversifying selection (red branches) in 6 branches within toxicofera. Alsi, *Alligator sinensis*; Cewa, *Celestus warreni*; Euma, *Eublepharis macularius*; Gein, *Gerrhonotus infernalis*; H_susp, *Heloderma suspectum*; Laag, *Lacerta agilis*; Pesi, *Pelodiscus sinensis*; Vaac, *Varanus acanthurus*; Vagi, *Varanus gilleni*; Vagl, *Varanus glauerti*; Vagig, *Varanus giganteus*; Vain, *Varanus indicus*; Vako, *Varanus komodoensis*; Vame, *Varanus mertens*; Vami, *Varanus mitchelli*; Vapa, *Varanus panoptes*; Vasc, *Varanus scalaris*. **B** Like in reptiles, venomous mammals experienced different selective pressures as compared to non-venomous mammals. aBSREL found evidence of diversifying selection one branch (red) leading up to a Solenodon copy (but see Supplementary Figure 11). Ereu, *Erinaceus europaeus*; Sopa, *Solenodon paradoxus*; BLTx, Blarina toxin; Calu, *Canis lupus*; Oran, *Ornithorhynchus anatinus*. **C** MEME identified 24 sites (in red) in the reptilian SVL that have experienced positive selection. Most of these sites are on the surface (raw output in supplementary dataset 9 and 10). These observations are consistent with previous estimates of high selection on surface residues of toxin serine protease [30]. **D** Unlike reptiles, however, only 10 sites on mammalian KLK1s showed evidence of positive selection, with a few on the surface.

We first used the branch-site unrestricted statistical test for episodic selection (BUSTED) to check for evidence of episodic diversifying selection on any site in the gene along any of the branches of toxicofera and venomous mammals [37]. For both mammals and reptiles, BUSTED found evidence for diversifying selection in at least one site on at least one test branch (Additional file 1: Fig.S7, Fig.S8). Since BUSTED revealed joint evidence of branch and site-specific selection, we used the adaptive branch site random effects model (aBSREL) and mixed effects model of evolution (MEME) to get a better resolution of positive selection in branches of the phylogeny and sites along the gene respectively [38, 39]. Testing the same toxicofera and venomous mammal lineages, aBSREL found evidence for episodic diversifying selection in 1 branch leading to one of the *Solenodon* KLK1 copies, while in toxicofera, it found evidence in 6 branches, one of them leading to the heloderma gilatoxin, another leading to a SVL copy in Haitian giant galliwasp (the lizard *Celestus warreni*), and the rest in branches leading up to the radiation of varanids (Fig. 2A, B).

The MEME model identified several sites in reptilian SVL genes and mammalian KLK genes that showed significant evidence of positive selection (*p* < 0.05). In reptile SVLs, MEME identified 24 sites experiencing positive selection, while in the mammalian KLKs, 10 sites were identified (Fig. 2C, D). While some of these sites were in the internal structure of the proteins, the majority of them were on surface residues.

We did not include the mouse-specific KLK1 in our main analyses as they are an expansion exclusive to mice and form a clade separate from the other mammalian KLKs, including those believed venomous in *Solenodon* and *Blarina* (Fig. 1B). However, for the sake of consistency, we performed selection tests using PAML, BUSTED, aBSREL, and MEME using the mouse-specific KLK1s. Overall, PAML, BUSTED, and MEME produced the same results as the previous analysis; venomous mammals experienced different rates of selection. In addition to evidence of selection along the same *Solenodon* branch, aBSREL found evidence along the blarina branch as well. The new results of selection analysis using the mouse sequences are found in the supplementary material (Additional file 1: Fig. S11, Fig. S12, Additional file 7-12). The large expansion of KLK1 in a lineage of mammals that are not venomous was fascinating. Using BUSTED and aBSREL, we tested for selection on venomous mammal lineages and the mouse expansion. Interestingly, both models found evidence of selection; BUSTED found evidence at the gene level and aBSREL showed evidence of selection in several specific mouse branches (Additional file 1: Fig. S13, Fig. S14). The functional relevance of this heightened selection is not clear, although there is evidence of sex-limited expression in mouse, suggesting a potential adaptive role in sex interactions [40].

## Discussion

Non-deterministic forces can give rise to evolutionary novelties de novo. Several well characterized mechanisms like gene duplication, gene fusion, and horizontal gene transfer are responsible for the birth of new genes [41]. These new genes in turn contribute to species specific processes and generate morphological and physiological diversity [42]. Although non-deterministic processes produce genetic variation (on which natural selection acts), many adaptive traits can be exapted through modifications of already pre-existing characters [43]. Such exaptation has led to the origin of vertebrate oral venoms on at least two levels. Recent work has shown that the ancestral salivary gland gene regulatory mechanisms were exapted in snake venom glands [44]. We now show that individual serine protease-based toxins used by diverse lineages share a common ancestor distinct from the ancestor of other non-toxin serine proteases. Thus, vertebrate venoms have evolved in parallel, at both the regulatory and also the genetic levels. This suggests that ancient shared history, namely salivary gland regulatory architecture and the presence of homologous genes biochemically suitable for toxicity, have facilitated venom evolution in distantly related taxa.

To determine the role of exaptation in venom evolution, it is important to understand the genetic makeup of adaptive traits, and how they lead to biochemical activity suitable for the envenomation. KLK1 genes in mammals and their reptilian homologs share kininogenase activity, which results in the release of bradykinin, a potent hypotensive agent, when injected into the bloodstream [23, 45]. This is true even of salivary kallikreins of non-venomous mammals, such as mice, which can induce hypotension and even death [46–48]. Hypotension is also one of two major strategies which venomous snakes use to immobilize their prey [49]. The biochemical link between bradykinin-producing enzymes in mammals and snakes was evident to researchers who first characterized kallikrein-like properties of a snake venom enzymes, calling them “the salivary kallikrein of the snake” [50]. That being said, biochemical similarity does not imply homology. Schachter [17] wrote in an early review that “kallikreins from different sources are not identical molecules, as originally assumed, nor is it likely that they are derived from a parent molecule”. While the biochemical homology of kallikrein venoms is now an accepted fact, the genetic homology and its role in the evolution of venoms was never extensively elaborated. Our analysis shows that genes underlying KLK-venom evolution in mammals and reptiles are homologous. Indeed, all KLK1 and SVL-like, and non-toxin KLK genes shared a common origin at the dawn of the tetrapods when they perhaps formed nearby gene clusters (Fig. 1A). However, even from within this family of paralogous proteases, venoms evolved from more closely related homologous genes as compared to the non-toxin KLKs (Fig. 1B).

### Evolution of tetrapod venoms by kallikrein exaptation

Most exaptations have bifunctional intermediates where both the old and new functions are preserved [51, 52]. This bifunctional nature likely allows for a gradual transition from one phenotypic state to another. For example, after gene duplication one or both the gene copies can perform its original function; or one copy can randomly acquire a new function in the course of accumulating neutral mutations [53]. This is the standard model of snake toxin evolution, which presuppose gene duplication prior to the acquisition of novel function (toxicity) [54, 55]. This is indeed observed in a recent study reconstructing the evolution of metalloproteinase toxins, which evolved from adam28 disintegrin by duplication and modification, such as the loss of a transmembrane domain improving solubility [19]. However, it appears that kallikreins already possess biochemical activity suitable for envenomation (vasodilation via bradykinin production); we have called such genes ‘toxipotent’. Interestingly, serine protease genes in viperid snake venoms have undergone extensive duplication, with no clear distinction (like the loss of disintegrin domain for SVMP, or deletion of PLA2 genes in viperids [19, 21]) between an ancestral gene and its derived toxic counterparts, which is at odds with the classical venom evolutionary model. However, while there does not seem to be substantial differences in nucleotide or amino acid sequences between the gene copies, variations in gene expression, protein expression, or biochemical activity might exist. So far, genomes of venomous mammals and *Heloderma* lizards are insufficiently well characterized to test whether a specific genetic modification(s) gave rise to the toxin serine proteases.

In a previous publication, we proposed a unified model of early venom evolution in mammals and reptiles, suggesting that venoms evolved when kallikreins already present in saliva increased (via higher copy number) and became more effective (via sequence level changes) [44]. In this study, we were able to reconstruct the evolution of ubiquitous kallikrein-based toxins via phylogenetics based on extensive taxonomic sampling and gene orthologs accurately selected from the wide range of serine proteases found in the genome based on phylogenetic and syntenic proximity. First, we found that copy number changes accompany the evolution of venom (e.g. snakes and *Solenodon*), but some lineages experience copy number expansions without evolving venom (mice and turtles, Fig. 1A). Second, we found that venomous taxa (Gila monster and *Solenodon*) indeed have a higher rate of nonsynonymous changes in the rates of venom evolution, consistent with selection for novel function (Fig. 2). Intriguingly, we also find evidence of selection in reptilian members of the Toxicofera clade, such as varanid lizards, where the existence of venom is debated (Fig. 2A) [56, 57]. From our results, the functional relevance of selection in the varanid lineage is not clear, though some studies have suggested a role of varanid oral secretions in prey procurement [29, 58, 59]. However, the presence of toxipotent genes in the saliva of many animals makes the line between venomous and non-venomous animals less clear. As most tetrapods already possess the requisite machinery for venom evolution, there could indeed be many taxa that lie on the continuum between what we currently perceive as venomous and non-venomous. Thus, the presence of serine proteases in saliva, and even sequence-level data suggesting past selection, may be insufficient to identify which animals are venomous. In order to do that, we need ecological evidence that animals, in fact, use their saliva for envenomation.

## Conclusion

**Fig. 3:**
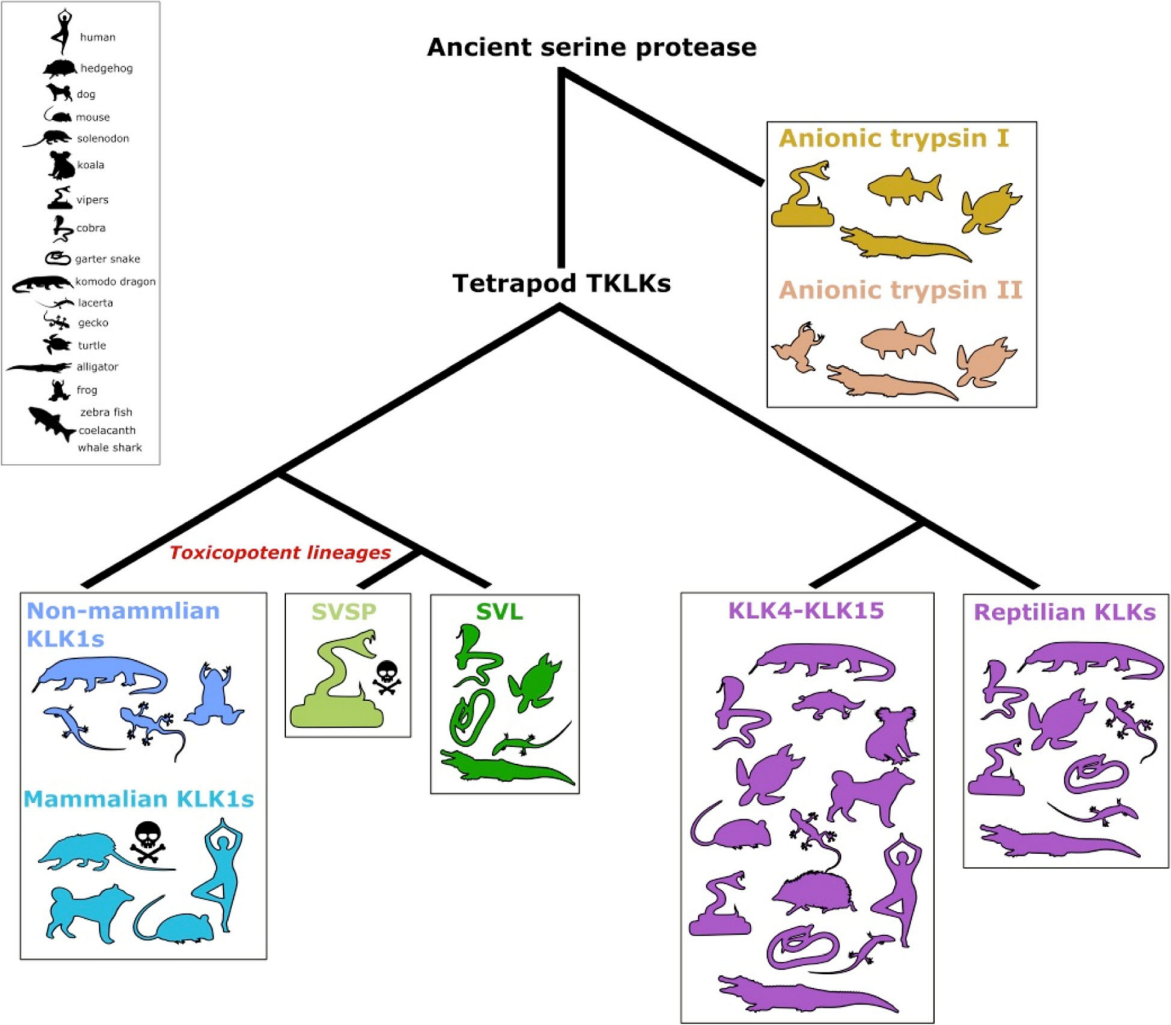
Evolution of tetrapod toxin kallikreins. The tetrapod lineage of TKLKs evolved from an ancestral serine protease that also gave rise to vertebrate anionic trypsins. From here, the tetrapod TKLKs diverged into the KLK4-KLK15 group and the toxicopotent KLK1-SVL-SVSP lineage. Proteins in KLK1-SVL-SVSP lineage are preadapted to become toxins, given their ability to regulate blood pressure when injected into the bloodstream

In this study, we expanded our knowledge on the phylogeny of kallikreins (KLKs) and, for the first time, with high certainty, resolved the relationship between tissue kallikreins (TKLKs) and their venomous counterparts in tetrapods. The tetrapod lineage of TKLKs evolved from an ancient serine protease that also gave rise to vertebrate anionic trypsins. From here, the tetrapod TKLKs diverged into the KLK4-KLK15 group and the toxicopotent KLK1-SVL-SVSP lineage (Fig. 3). These toxicopotent homologs eventually diversified and became a part of venom in snakes, some lizards, as well as some shrews and solenodon. We add to a long held belief that venoms primarily originate through a combination of constraint and convergence and show that shared history and parallel evolution (parallelism) can explain the repeated evolution of toxins in venoms. Parallelism is sometimes considered a process that led to the rise of phenotypic similarity in closely related species [8]. While this perspective can account for a shared molecular basis and history, the numerous exceptions to this prevents it from being definitive [60, 61]. It is more appropriate to consider parallelism as the use of shared molecular mechanisms to produce convergent phenotypes, irrespective of their taxonomic proximity [62]. We illustrate this by showing that venom in mammals and reptiles originated multiple times in parallel by modifying the same gene family despite 300 million years separating these lineages. Thus, ancient conserved molecular mechanisms and building blocks can continue to be a source of adaptive novelty, allowing nature to replay the tape of life, albeit with a new perspective.

## Methods

### Genomic analysis

We used publicly available vertebrate genomes of good quality (Additional file 13) to establish location and synteny of the Kallikrein clusters. We used genomes for which RNA-seq verified genomic annotations were available as a reference point and created an extensive map of the genes that flank SVL and TKL in those genomes. These include HPN, SCN1B, GRAMD1A, PSMC4 RBM42, HAUS5, and MAG (Additional file 1: Fig.S1, Fig.S2). That allowed us to establish syntenic relationships of those regions in different genomes. We then proceeded to use those flanking genes as a database to BLAST (NCBI-BLAST v.2.7.1+ suite, blastn, e-value cutoff of 0.05, default restrictions on word count and gaps) the genomes if they were less well annotated. That gave us a number of genomic scaffolds that potentially contained KLK genes. We used those for the second round of BLAST (tblastx, e-value cutoff of 0.01) against a database of exons extracted from well-annotated mammalian TKL and viper SVL genes. Positive hits were checked by eye in Geneious v11 (https://www.geneious.com), and any complete exons were manually annotated and later merged into CDS of newly annotated genes if the exon order and count was in accordance with existing reliable KLK annotations. All resulting genes that produced viable mature peptides were then used for the phylogenetic analysis.

### Phylogenetic analysis

All viable genes located in the previous step were translated into proteins and aligned with selected publicly available sequences of interest using L-INS-i method of MAFFT software v7.305 (Katoh and Standley 2013) with 1000 iterations (--localpair --maxiterate 1000). These parameters were used for all subsequent alignments. The publicly available serine protease sequences were obtained from NCBI. Using human KLK1 (gene ID: 3816) as a search query we obtained a list of all similar genes that were estimated based on synteny information and conserved protein domains. We selected sequences from Human (*Homo sapiens*), mouse (*Mus musculus*), dog (*Canis lupus familiaris*), hedgehog (*Erinaceus europaeus*), Lacerta (*Lacerta agilis*), garter snake (*Thamnophis elegans*), habu (*Protobothrops mucrosquamatus*), Chinese soft-shell turtle (*Pelodiscus sinensi*s), alligator (*Alligator sinensis*), frog (*Xenopus tropicalis*), zebra fish (*Danio rerio*), coelacanth (*Latimeria chalumnae*), and whale shark (*Rhincodon typus*). These gene sets were used as input for OrthoFinder (OF). Using an mcl threshold of 1.2 OF grouped closely related genes into several orthogroups. We selected the orthogroup that contained SVSP-SVL-KLK1 sequences for a more rigorous phylogenetic analysis (Additional file 2). We selected complement D and granzyme (which were not present in the orthogroup mentioned above) as outgroups. Alignments were observed in Geneious v11 (https://www.geneious.com). As a sanity check, we made sure that known homologous parts of the molecule (like the cysteine backbone which is a prominent, highly conserved feature of serine proteases [30]) were aligned properly. A final alignment with 50% masked gaps was used to make the tree (Additional file 14). We constructed the Bayesian Phylogeny using MrBayes (v3.2.3) [63]. The analysis used a mixed amino acid model and was carried out across two parallel runs for 200 million generations [64], by which point the standard deviation of split frequencies reached 0.0065. Half of the trees were removed as burn-in and the rest summarized to compute posterior probabilities. We also computed Bayes factor support for monophyly of SVSPs and KLK1/2/3 vs. the monophyly of all KLK genes by stepping-stone sampling of tree space with corresponding backbone constraints for 50 million generations [65]. The maximum-likelihood phylogeny was constructed using PhyML (v3.3.2) [66]. PhyML selected the WAG +G+I model based on Akaike Information Criteria [67]. Branch supports were calculated using aBayes [68]. aBayes is a Bayesian-like transformation of approximate likelihood-ratio test (aLRT) that offers the highest power compared to other methods to estimate node support and values that have similar interpretation to Bayesian posterior probabilities [68].

### Selection analysis

Alignments for sequence analysis were carried out using the MAFFT alignment tool, implementing the E-INS-i algorithm with BLOSUM62 as the scoring matrix [69]. All alignments were trimmed to remove signal peptide. The phylogeny was constructed based on a neighbour-joining tree using the Jukes-Cantor model. Additional anguimorpha kallikrein can be found in (Additional file 15). To test for selection on branches leading to venomous animals we used maximum likelihood models implemented in CodeML of the PAML package [33]. The log likelihood was compared between test branches (venomous animals) vs. background branches (non-venomous animals), and significant difference in models was determined using a log likelihood ratio test. Tests for adaptive evolution using BUSTED, aBSREL, and MEME analysis were carried out on the Datamonkey server [36]. The three-dimensional protein models for SVL and KLK1 were generated using a homology search implemented on the Phyre2 server [70] using consensus sequences obtained from the alignment of reptile SVLs and mammalian KLKs used in the selection analysis. PyMOL was used for visualization (PyMOL Molecular Graphics System, Schrӧdinger, LLC).

## Supplementary Information

**Additional file 1: Figures S1-S14.** **Fig. S1**- Synteny of SVSP and SVL genes. **Fig. S2**- Synteny of Kallikreins across mammals and reptiles. **Fig. S3**- Bayesian phylogeny of SVL-SVSP-KLKs. **Fig. S4**- Additional anguimorpha kallikrein sequences. **Fig. S5**- Test and background branches for reptiles. **Fig. S6**- Test and background branches for mammals. **Fig. S7**- Evidence ratio for BUSTED model in reptiles. **Fig. S8**- Evidence ratio for BUSTED model in mammals. **Fig. S9**- Phylogenetic tree showing aBSREL result for branch specific selection in reptiles. **Fig. S10**- Phylogenetic tree showing aBSREL result for branch specific selection in mammals. **Fig. S11**- Evidence ratio for BUSTED model in mouse KLK copies analysis. **Fig. S12**- aBSREL result in mouse KLK copies analysis. **Fig. S13**- Evidence ratio for BUSTED model in venomous mammals with mouse KLK copies. **Fig. S14**- aBSREL result in venomous mammals with mouse KLK copies.

**Additional file 2.** – List of orthogroups.

**Additional file 3.** – PhyML tree file.

**Additional file 4.** – MrBayes tree file.

**Additional file 5.** – PAML results reptiles.

**Additional file 6.** – PAML results mammals.

**Additional file 7.** – BUSTED results mouse KLK copies.

**Additional file 8.** – aBSREL results mouse KLK copies.

**Additional file 9.** – MEME results mouse KLK copies.

**Additional file 10.** – PAML mouse KLK copies H1 test.

**Additional file 11.** – PAML mouse KLK copies H0 null.

**Additional file 12.** – Alignment of mouse KLK copies and other sequences.

**Additional file 13.** – Genome accessions.

**Additional file 14.** – Alignment of all sequences for main phylogeney.

**Additional file 15.** – Anguimorpha sequences.

**Additional file 16.** – BUSTED results reptiles.

**Additional file 17.** – BUSTED results mammals.

**Additional file 18.** – aBSREL results reptiles.

**Additional file 19.** – aBSREL results mammals.

**Additional file 20.** – MEME results reptiles.

**Additional file 21.** – MEME results mammals.

**Additional file 22.** – BUSTED results venomous mammals and mouse KLK copies.

**Additional file 23.** – aBSREL results venomous mammals and mouse KLK copies.

**Additional file 24.** – PhyML stats and standard out file.

## Data Availability

All data including sequences and output of phylogenetic programs is included within the article and its additional files.

## Abbreviations

aBSREL: Adaptive branch site random effects likelihood
BUSTED: Branch-site unrestricted statistical test for episodic selection
KLK: Kallikreins
LRT: Likelihood-ratio test
MEME: Mixed effects model of evolution
mKLK1,2,3: Mammalian kallikreins 1, 2, and 3
PAML: Phylogenetic analysis using maximum likelihood
SVL: Snake-venom like
SVSP: Snake venom serine protease
TKLs: Tissue kallikreins
