# Supplementary material for: Co-option of the same ancestral gene family gave rise to mammalian and reptilian toxins
Source: BMC Biol. 2021 Dec 23;19:268. doi: 10.1186/s12915-021-01191-1 (PMC8705180; doi:10.1186/s12915-021-01191-1)
Supplement: Supplementary file 1 — Additional file 1: Figures S1-S14. Fig. S1- Synteny of SVSP and SVL genes. Fig. S2- Synteny of Kallikreins across mammals and reptiles. Fig. S3- Bayesian phylogeny of SVL-SVSP-KLKs. Fig. S4- Additional anguimorpha kallikrein sequences. Fig. S5- Test and background branches for reptiles. Fig. S6- Test and background branches for mammals. Fig. S7- Evidence ratio for BUSTED model in reptiles. Fig. S8- Evidence ratio for BUSTED model in mammals. Fig. S9- Phylogenetic tree showing aBSREL result for branch specific selection in reptiles. Fig. S10- Phylogenetic tree showing aBSREL result for branch specific selection in mammals. Fig. S11- Evidence ratio for BUSTED model in mouse KLK copies analysis. Fig. S12- aBSREL result in mouse KLK copies analysis. Fig. S13- Evidence ratio for BUSTED model in venomous mammals with mouse KLK copies. Fig. S14- aBSREL result in venomous mammals with mouse KLK copies. [file 12915_2021_1191_MOESM1_ESM.pdf]

Supplementary material for: Co-option of the same ancestral gene family gave rise to mammalian and reptilian toxins

Agneesh Barua[1,+], Ivan Koludarov[1,2,+], Alexander S. Mikheyev[1,3,*]

+Co-first author

*Corresponding author

1. Ecology and Evolution Unit, Okinawa Institute of Science and Technology Graduate University, Okinawa, Japan
2. Animal Venomics Group, Justus Leibig University, Giessen, Germany
3. Research School of Biology, Australian National University, Canberra, ACT, Australia

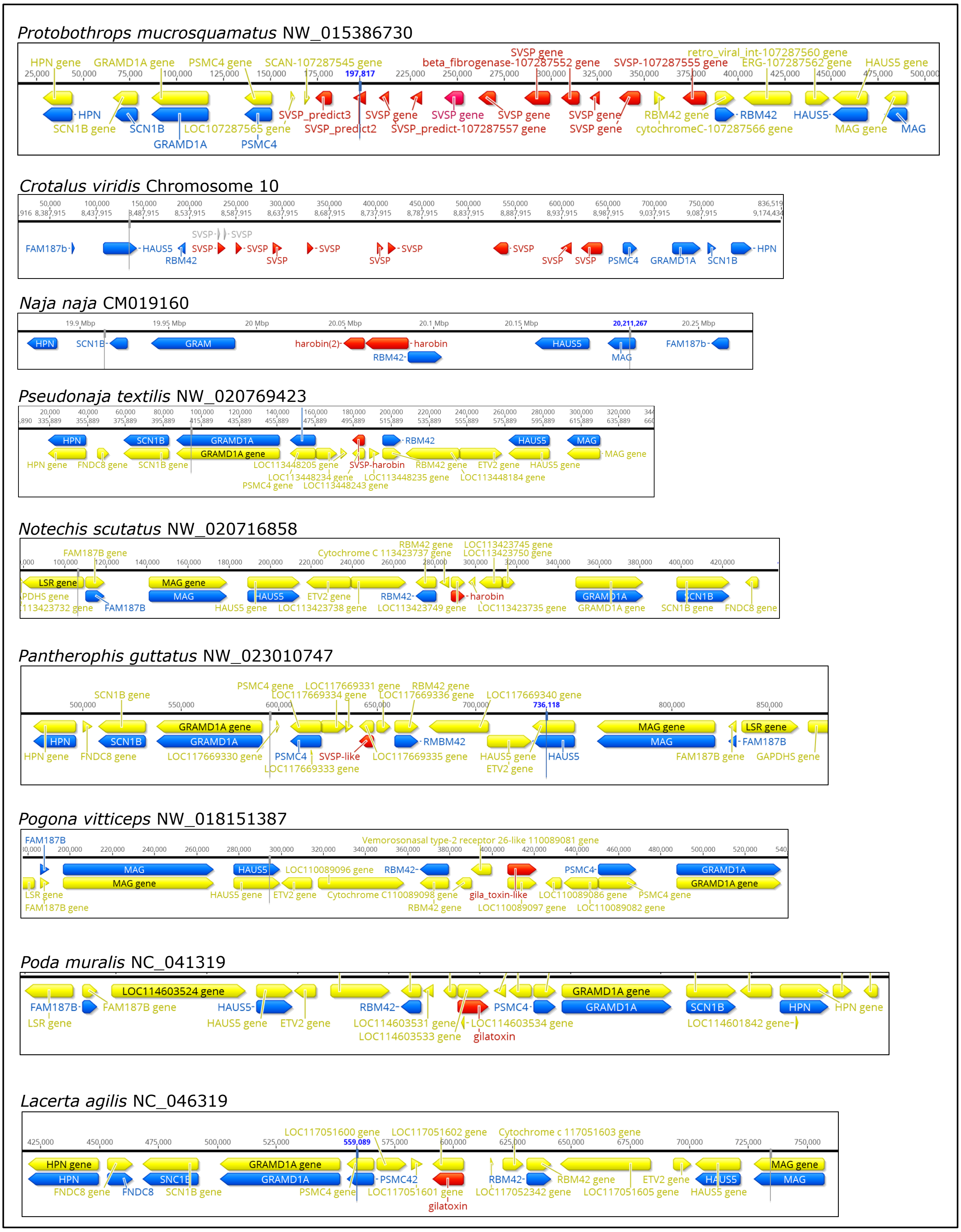

Figure S1: Synteny of SVSP and SVL genes. A group of reptilian KLKs (orange) are highly syntenic with snake venom serine protease (SVSP) from vipers. Above figure shows scaffolds that contain SVSP and Snake Venom Like (SVL) serine proteases in reptiles. Throughout the annotations in different genomes these genes were referred to by different names. For consistency we refer to them as SVL. The blue gene models are manual annotations that were created to help in pinpointing the region of interest

in the entire scaffold. The yellow annotations are obtained from GeneBank files. For the scaffolds that did not have GeneBank annotations (Naja and Crotalus) manual annotations (blue) were used. The above visualization was done in Geneiousv11 (https://www.geneious.com).

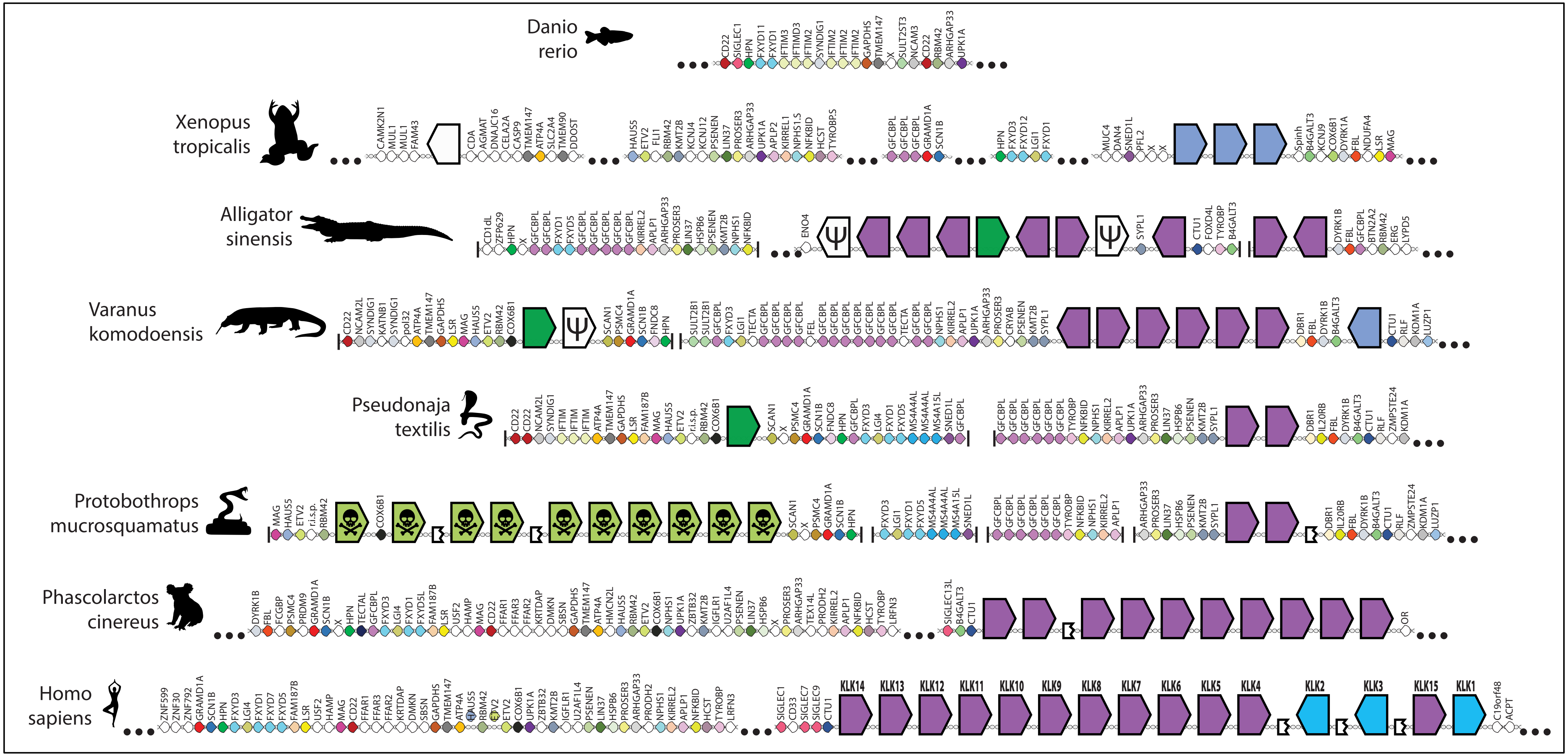

Figure S2: Synteny of Kallikreins across mammals and reptiles. Existing genomic assemblies of vertebrates reveal stable synteny of tissue kallikreins (TKL). Big arrow boxes represent individual TKL genes, color-coded consistently with the main manuscript, greek letter *psi* designates pseudogenes among them. Incomplete genes and stray exons are represented by white boxes with a single jagged side.

All non-TKL genes are represented by small pointed discs colored by homology and labelled according to their most commonly used abbreviation, white coloring designates unique genes. Black vertical lines indicate break in bioinformatic scaffolding, three black dots indicate long continuous streaks of genomic regions.

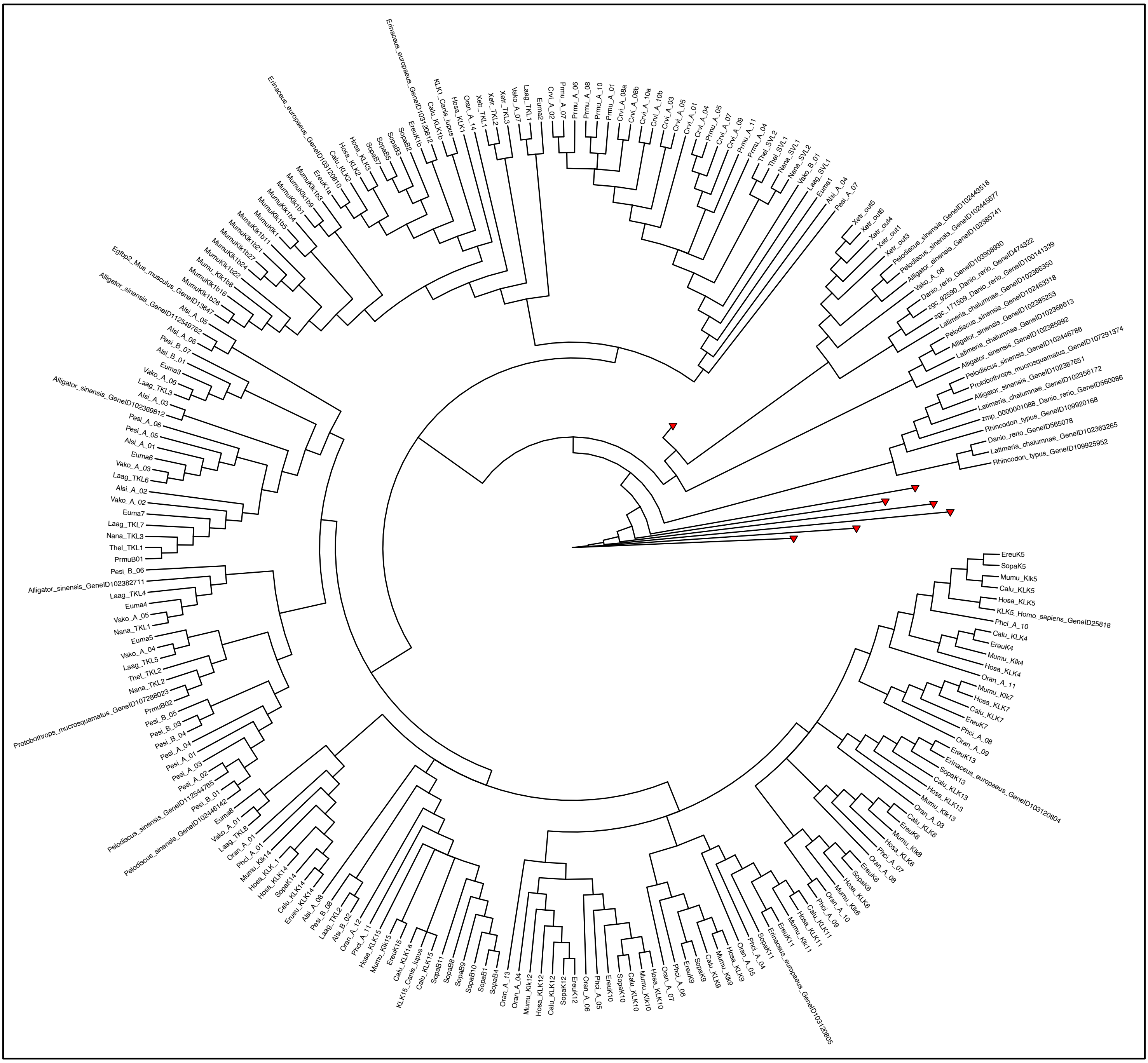

Figure S3: Bayesian phylogeny of SVL-SVSP-KLKs. Phylogenetic analysis revealed that tetrapod TKLs originated from a common ancestor with vertebrate anionic trypsins, which are commonly expressed in the pancreas and are found elsewhere in the genome. TKLs diverged into two distinct clades, one comprising the KLK4-KLK15 lineages and the other the KLK1/2/3-SVSP/SVL lineage that contains toxipotent genes. Red tips represent collapsed nodes. Raw tree file can be found in supplementary dataset 1.

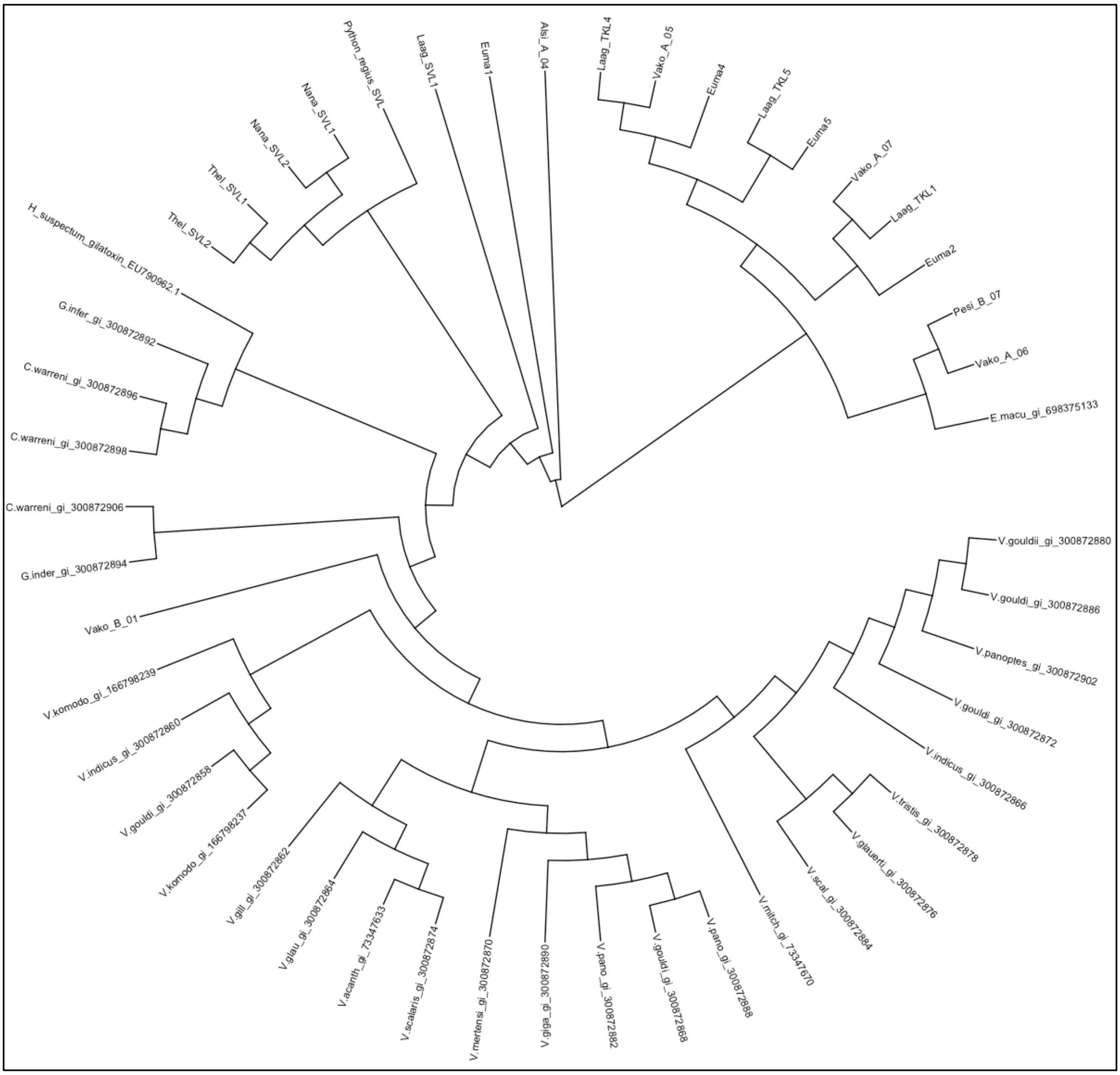

Figure S4: Additional anguimorpha kallikrein sequences were obtained from NCBI and aligned using MAFFT aligner. Laag_TKL4, VakoA05, Euma4, LaagTKL5, Euma5, VakoA07, LaagTKL1, Euma2, Vaki_A06, Emau_gi were not used in the selection analysis as they represent a separate clade from that of the snake venom like KLKs. Pesi_B7 was used as an outgroup. Alsi: *Alligator sinensis*, Cewa: *Celestus warreni*, Euma: *Eublepharis macularius*, Gein: *Gerrhonotus infernalis*, H_susp: *Heloderma suspectum*, Laag: *Lacerta agilis*, Pesi: *Pelodiscus sinensis*, Vaac: *Varanus acanthurus*, Vagi: *Varanus gilleni*, Vagl: *Varanus glauerti*, Vagig: *Varanus giganteus*, Vain: *Varanus indicus*, Vako:

Varanus komodoensis, Vame: *Varanus mertens*, Vami: *Varanus mitchelli*, Vapa: *Varanus panoptes*, Vasc: *Varanus scalaris*.

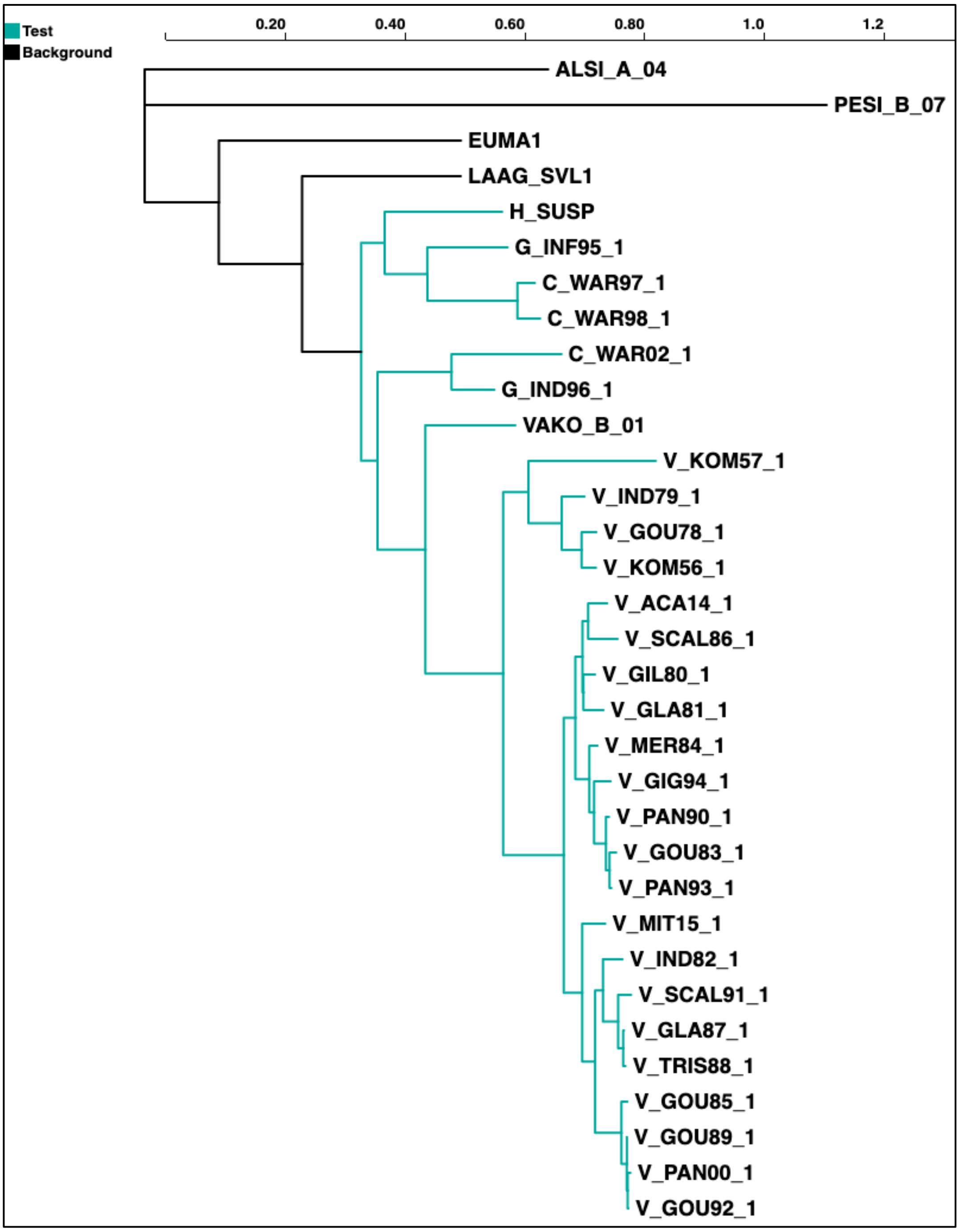

Figure S5: Test and background branches in reptiles used for all phylogenetic hypothesis tests (PAML, BUSTED, aBSREL).

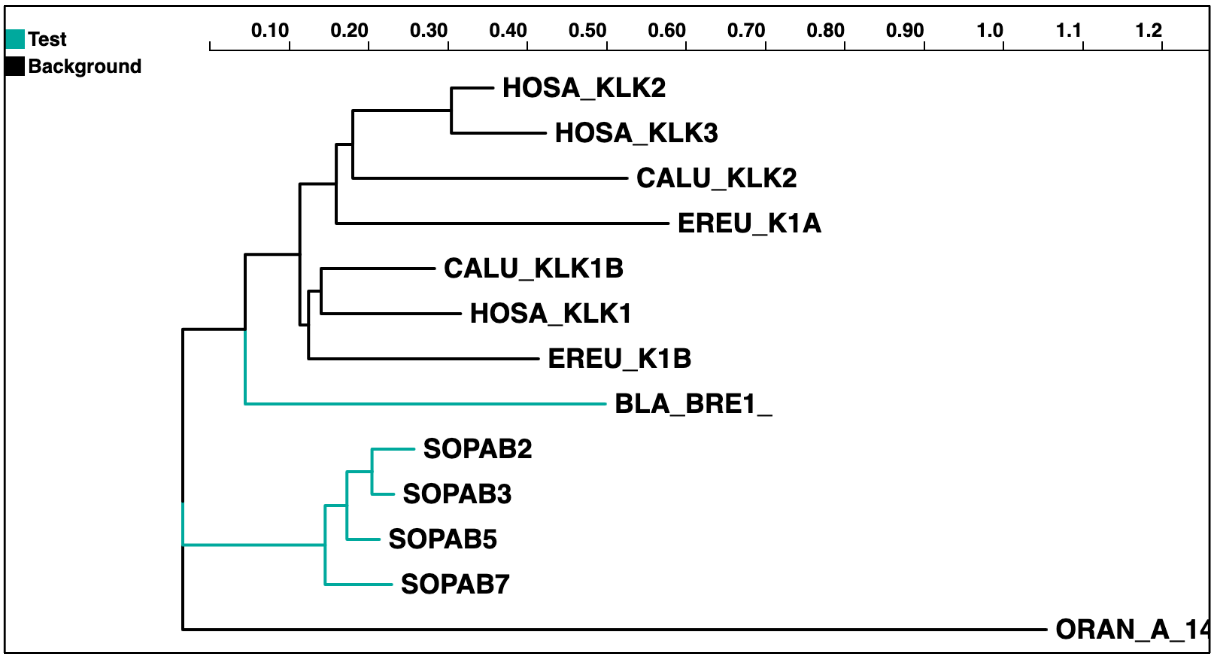

Figure S6: Test and background branches in mammals used for all phylogenetic hypothesis tests (PAML, BUSTED, aBSREL).

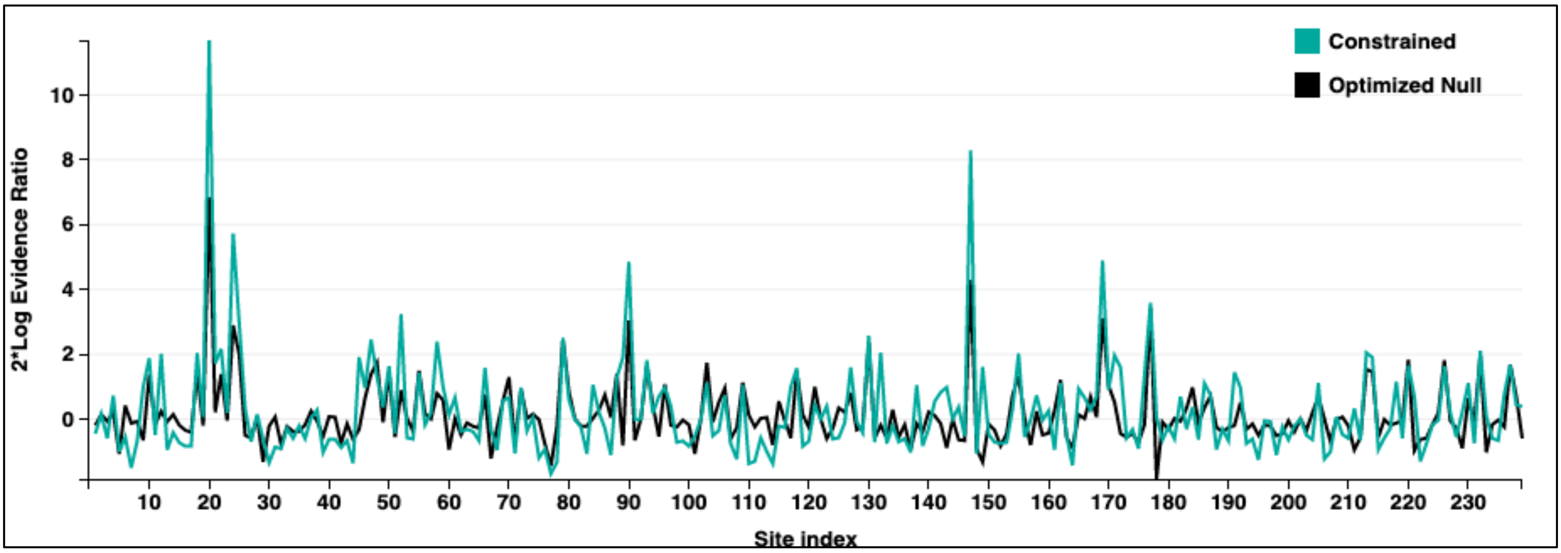

Figure S7: Evidence ratio for BUSTED model in reptiles. BUSTED with synonymous rate variation found evidence (LRT, p-value = 0.000 ≤ .05) of gene-wide episodic diversifying selection in the selected test branches in the reptile phylogeny. Therefore, there is evidence that at least one site on at least one test branch has experienced diversifying selection. The Evidence Ratio (y-axis) gives the likelihood ratio (on a log-scale) that the alternative model (selection along test branches) was a better fit to the data as compared to the null model. JSON file containing raw output of BUSTED can be found in supplementary dataset 5.

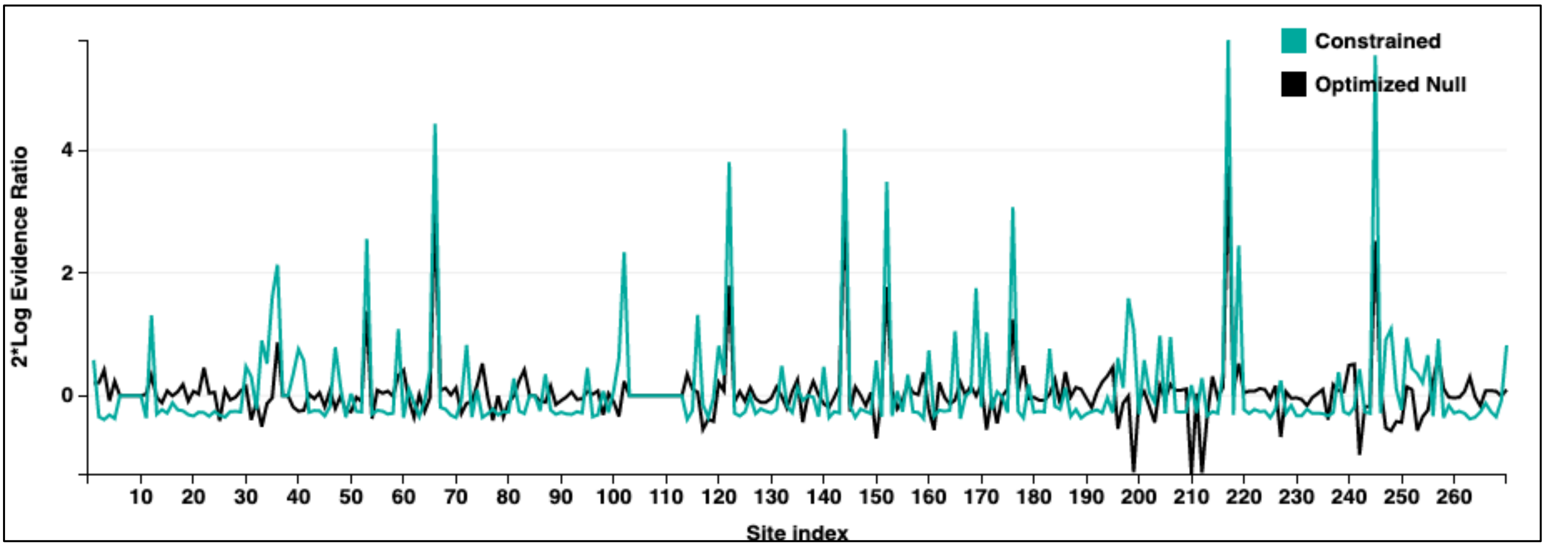

Figure S8: Evidence ratio for BUSTED model in mammals. BUSTED with synonymous rate variation found evidence (LRT, p-value = 0.005 ≤ .05) of gene-wide episodic diversifying selection in the selected test branches in the mammal phylogeny. Therefore, there is evidence that at least one site on at least one test branch has experienced diversifying selection. The Evidence Ratio (y-axis) gives the likelihood ratio (on a log-scale) that the alternative model (selection along test branches) was a better fit to the data as compared to the null model. JSON file containing raw output of BUSTED can be found in supplementary dataset 6.

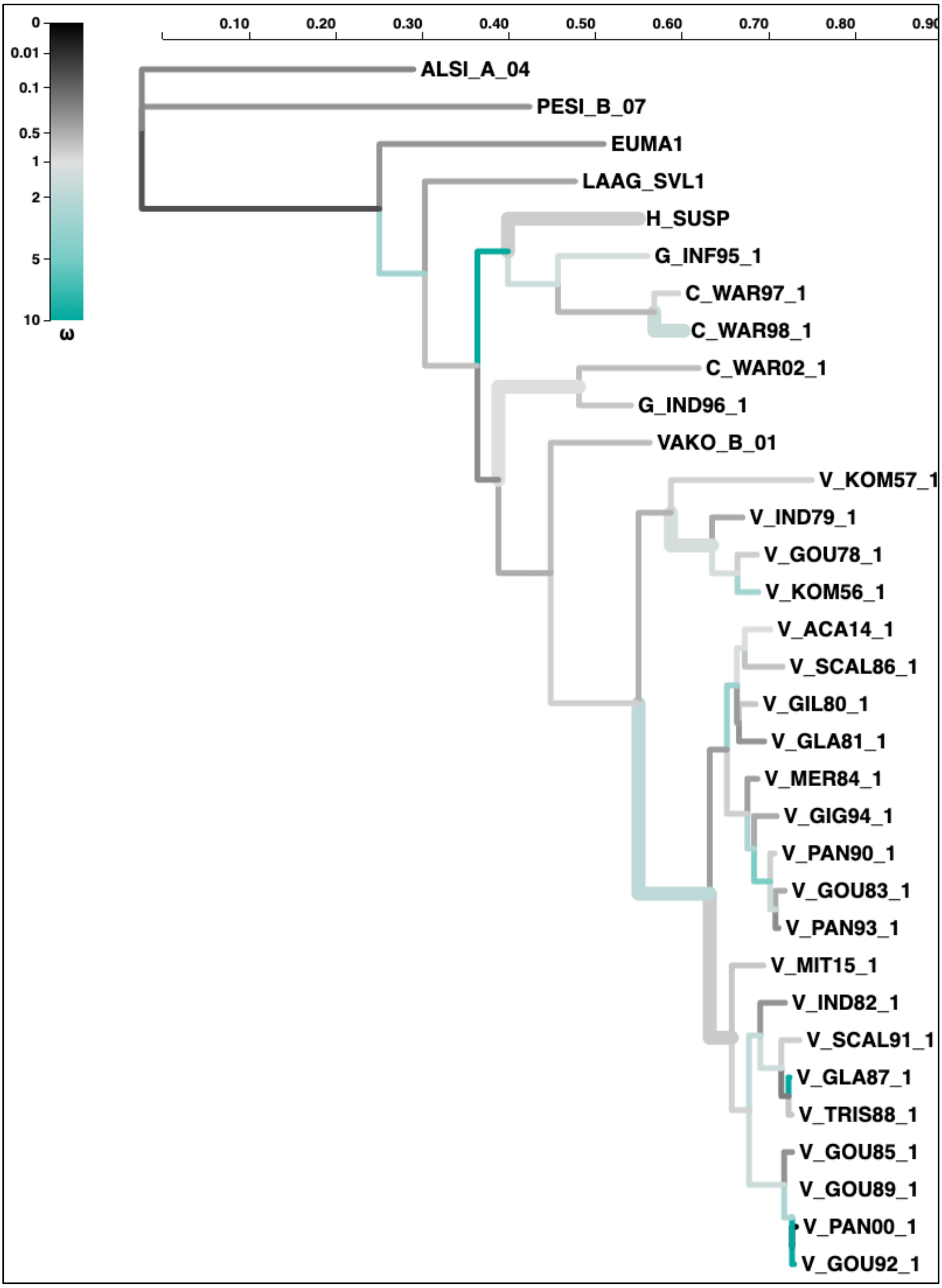

Figure S9: Phylogenetic tree showing aBSREL result for branch specific selection in reptiles. Only the thick branches represent significant evidence for diversifying selection. JSON file of raw output is in supplementary dataset 7.

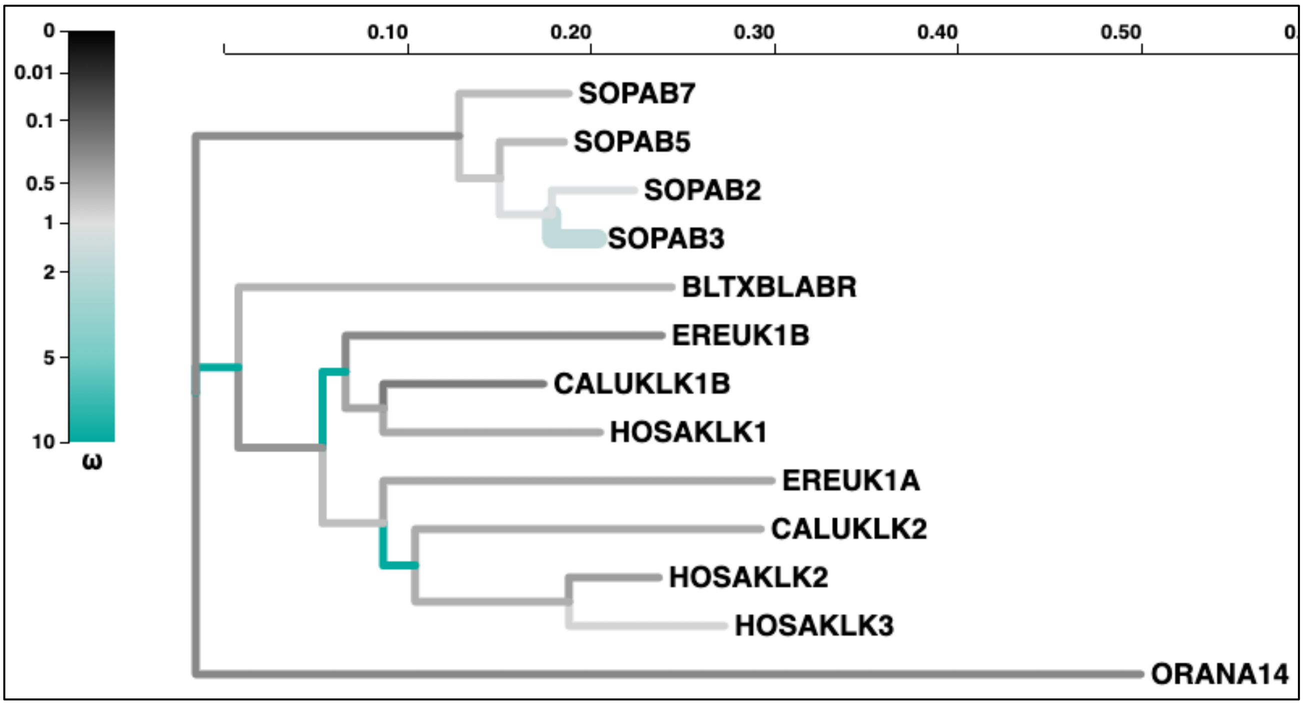

Figure S10: Phylogenetic tree showing aBSREL result for branch specific selection in mammals. Only the thick branches represent significant evidence for diversifying selection. JSON file of raw output is in supplementary dataset 8.

Selection analysis with mouse KLK1 expansions.

Our aim for this analysis was to test whether the KLK genes in venomous lineages like *Solenodon* and *Blarina* experienced different rates of selection. In this regard, adding the mouse sequences would only increase the amount of background information (since for any branch-level test single derives from a single branch). Nonetheless, we ran the analysis using the mouse sequences. The PAML results are unchanged; the venomous lineage of *Solenodon* and *Blarina* experienced selective forces significantly different (LRT, $p \leq 0.05$) from the rest of the tree (Supplementary datasets 20, 21).

The results of BUSTED and MEME with and without mouse sequences are not directly comparable because the number of sequences (27 as opposed to 13) and sites (295 as opposed to 270) changes between the two data sets. Therefore, their model parameter estimates will be different and model fit cannot be judged under the same criteria. Despite that, the overall results for BUSTED, and MEME are the same with evidence of sites experiencing significant selection.

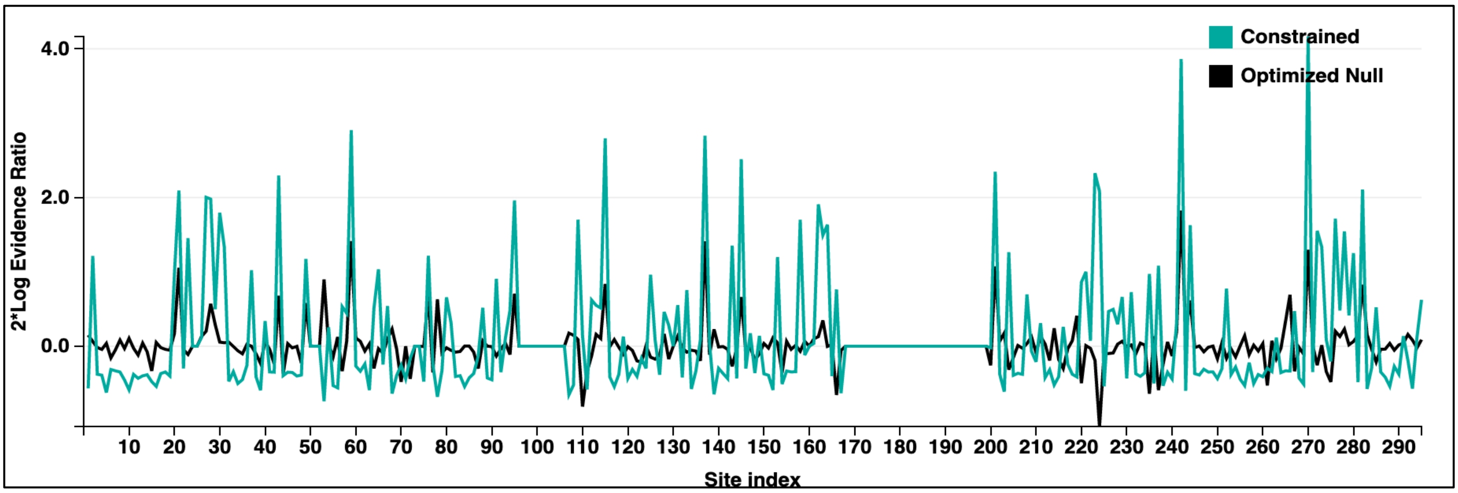

Figure S11: Evidence ratio for BUSTED model in mammals with mouse KLK1 copies. BUSTED with synonymous rate variation found evidence (LRT, p-value = 0.029 ≤ .05) of gene-wide episodic diversifying selection in the selected test branches in the phylogeny. Therefore, there is evidence that at least one site on at least one test branch has experienced diversifying selection. The Evidence Ratio (y-axis) gives the likelihood ratio (on a log-scale) that the alternative model (selection along test branches) was a better fit to the data as compared to the null model. JSON file containing raw output of BUSTED can be found in supplementary dataset 13.

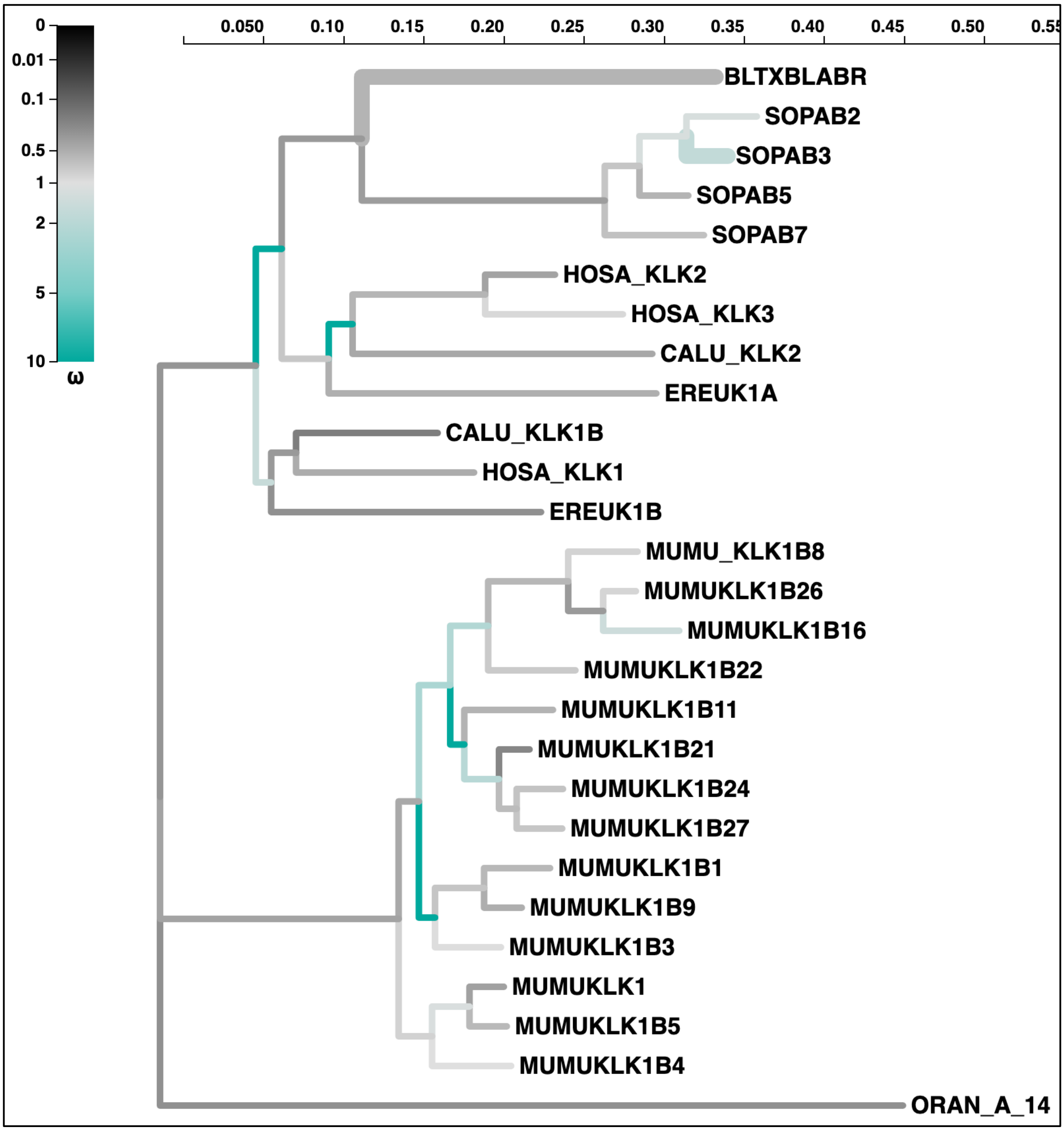

Figure S12: Phylogenetic tree showing aBSREL result for branch specific selection in mammals with mouse copies. With the inclusion of mouse sequences, aBSREL found evidence of selection along the same *Solenodon* branch. Additionally, it also found evidence along the branch leading to the *Blarina* sequence. This result was reassuring as both these mammals are considered venomous and evidence of selection in their venom was expected. Only the thick branches represent significant evidence for diversifying selection. JSON file of raw output is in supplementary dataset 14.

The large expansion of KLK1 in a lineage of mammals that are not venomous was fascinating. Using BUSTED and aBSREL, we tested for selection on venomous mammal lineages and the mouse expansion. Interestingly, both models found evidence of selection; BUSTED found evidence at the gene level and aBSREL showed evidence of selection in several specific mouse branches.

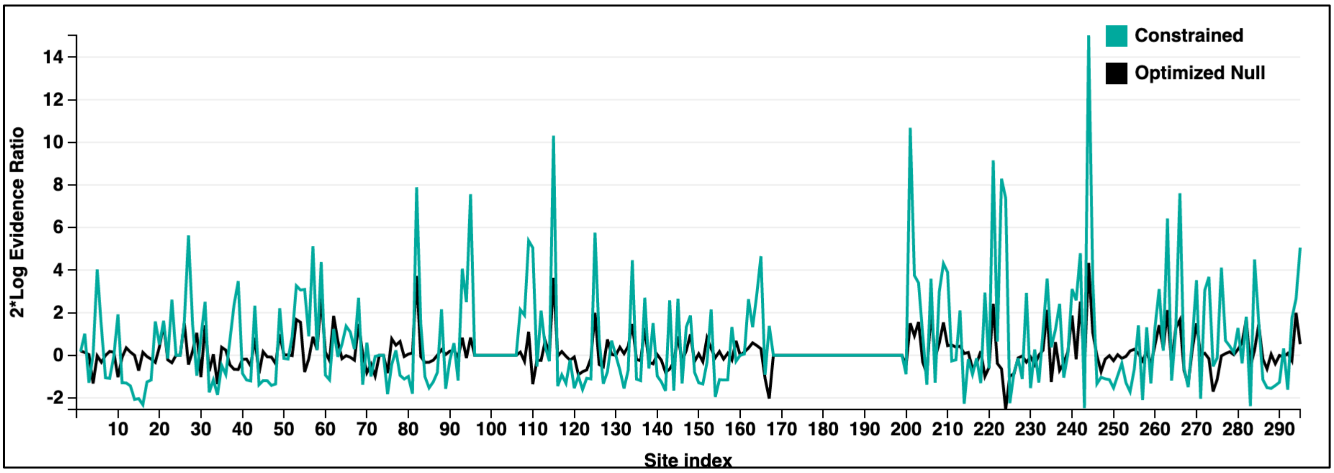

Figure S13: Evidence ratio for BUSTED model in venomous mammals with mouse KLK1 copies as test branches. BUSTED with synonymous rate variation found evidence (LRT, p-value = 0.000 ≤ .05) of gene-wide episodic diversifying selection in the selected test branches in the phylogeny. Therefore, there is evidence that at least one site on at least one test branch has experienced diversifying selection. The Evidence Ratio (y-axis) gives the likelihood ratio (on a log-scale) that the alternative model (selection along test branches) was a better fit to the data as compared to the null model. JSON file containing raw output of BUSTED can be found in supplementary dataset 16.

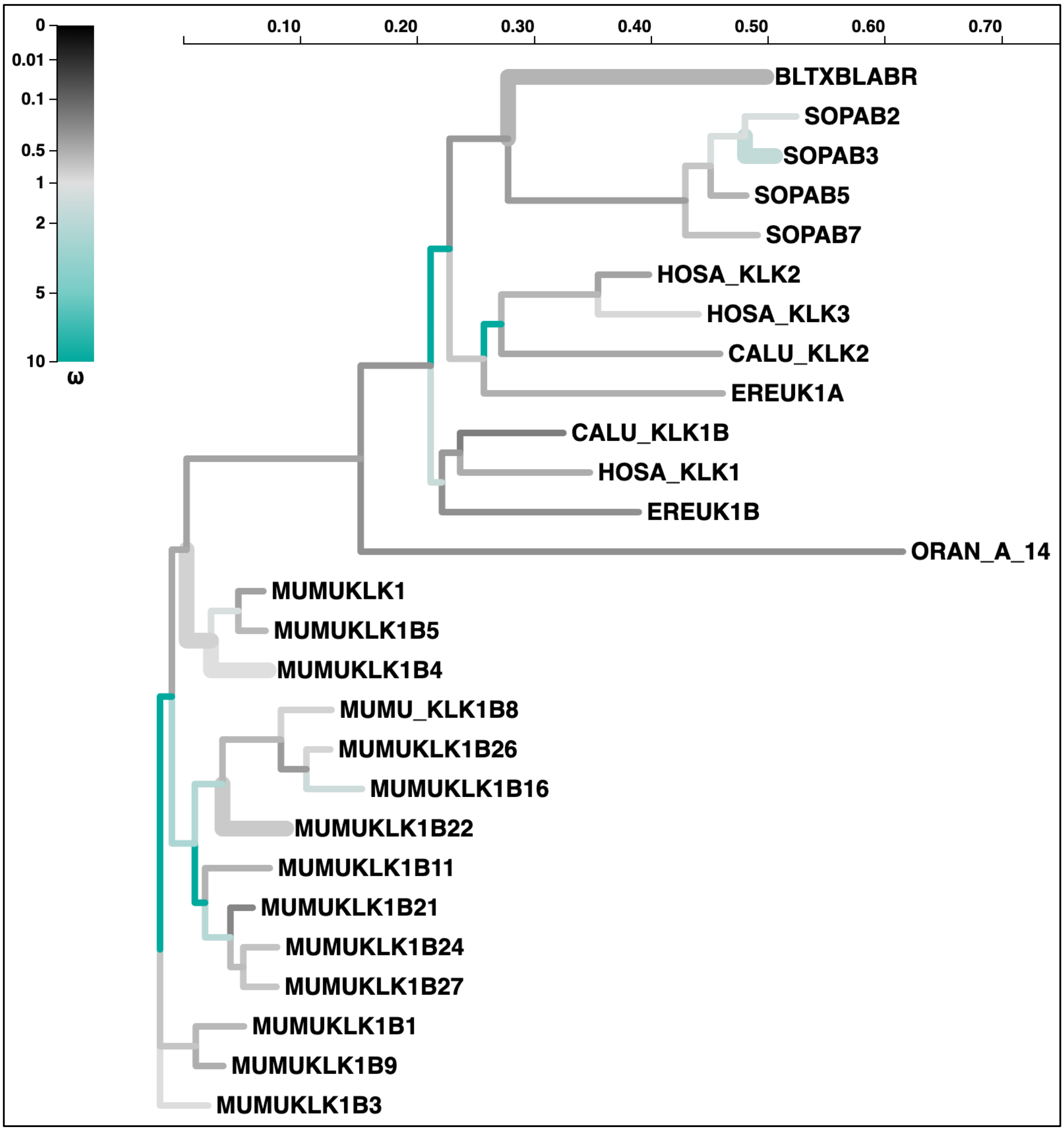

Figure S14: Phylogenetic tree showing aBSREL result for branch specific selection in venomous mammals and mouse KLK1 copies. aBSREL found evidence of selection along some KLK1 branches in mouse. Only the thick branches represent significant evidence for diversifying selection. JSON file of raw output is in supplementary dataset 17.
